# An MMP13-Selective Inhibitor Delays Primary Tumor Growth and the Onset of Tumor-Associated Osteolytic Lesions in Experimental Models of Breast Cancer

**DOI:** 10.1371/journal.pone.0029615

**Published:** 2012-01-11

**Authors:** Manisha Shah, Dexing Huang, Tony Blick, Andrea Connor, Lawrence A. Reiter, Joel R. Hardink, Conor C. Lynch, Mark Waltham, Erik W. Thompson

**Affiliations:** 1 St. Vincent's Institute of Medical Research, St. Vincent's Hospital, Melbourne, Australia; 2 University of Melbourne Department of Surgery, St. Vincent's Hospital, Melbourne, Australia; 3 Pfizer Global Research and Development, Groton Laboratories, Groton, Connecticut, United States of America; 4 H. Lee Moffitt Cancer Center and Research Institute, Tampa, Florida, United States of America; Stanford University, United States of America

## Abstract

We investigated the effects of the matrix metalloproteinase 13 (MMP13)-selective inhibitor, 5-(4-{4-[4-(4-fluorophenyl)-1,3-oxazol-2-yl]phenoxy}phenoxy)-5-(2-methoxyethyl) pyrimidine-2,4,6(1H,3H,5H)-trione (Cmpd-1), on the primary tumor growth and breast cancer-associated bone remodeling using xenograft and syngeneic mouse models. We used human breast cancer MDA-MB-231 cells inoculated into the mammary fat pad and left ventricle of BALB/c Nu/Nu mice, respectively, and spontaneously metastasizing 4T1.2-Luc mouse mammary cells inoculated into mammary fat pad of BALB/c mice. In a prevention setting, treatment with Cmpd-1 markedly delayed the growth of primary tumors in both models, and reduced the onset and severity of osteolytic lesions in the MDA-MB-231 intracardiac model. Intervention treatment with Cmpd-1 on established MDA-MB-231 primary tumors also significantly inhibited subsequent growth. In contrast, no effects of Cmpd-1 were observed on soft organ metastatic burden following intracardiac or mammary fat pad inoculations of MDA-MB-231 and 4T1.2-Luc cells respectively. MMP13 immunostaining of clinical primary breast tumors and experimental mice tumors revealed intra-tumoral and stromal expression in most tumors, and vasculature expression in all. MMP13 was also detected in osteoblasts in clinical samples of breast-to-bone metastases. The data suggest that MMP13-selective inhibitors, which lack musculoskeletal side effects, may have therapeutic potential both in primary breast cancer and cancer-induced bone osteolysis.

## Introduction

The human matrix metalloproteinase (MMP) family comprises 26 zinc-dependent transmembrane and secreted neutral endopeptidases that contribute to homeostasis of the extracellular matrix [1]. MMPs are involved in a variety of physiological and pathological signaling processes characterized by tissue destruction, including arthritis, atherosclerosis and cancer. These proteases have been implicated in multiple facets of tumorigenesis, including primary tumor growth, angiogenesis, local invasion/migration, intravasation and extravasation, and also in the establishment and growth of metastatic lesions [1].

MMPs are synthesized by tumor cells, but predominantly produced by the surrounding stromal cells [2]. Important anti-tumoral roles for several MMPs have been revealed, and these may partly explain the failure of early MMP inhibitor trials [3]. In addition, early clinical trials were hampered by a lack of efficacy markers to guide dosing, and by dose-limiting toxicity such as musculoskeletal syndrome (MSS), which is characterized by painful stiffening of joints, tendonitis, soft tissue fibroplasias and inflammation [3]. The development of agents that specifically inhibit individual MMPs associated with particular cancers is postulated to provide effective therapeutics that may overcome these problems [4].

MMP13 may be such a candidate. Its selective inhibition may be clinically beneficial over the relatively non-selective broad-spectrum MMP inhibitors based on the fact that MMP13 expression is largely restricted to pathological conditions including various carcinomas. For example, elevated levels of MMP13 have been associated with decreased overall survival and lymph node metastasis in breast cancer [5], bone metastasis in renal cell carcinoma [6], poor prognosis of non-small cell lung and colorectal cancers [7], [8] and invasive capability in various other human cancers including melanoma, head and neck and vulvar squamous cell carcinoma [9]. Furthermore, stroma-derived MMP13 was recently found to be involved in the growth and organ-specific metastasis of melanoma [10]. We have previously shown that MMP13 was dramatically induced in the tumor-associated stroma of human breast cancer xenografts [11].

MMP13 has an important role in bone remodeling [12], [13], and in bone cancer and cancers that frequently metastasize to bone, such as breast and prostate cancer and multiple myeloma [14], [15], [16]. In particular, a recent finding demonstrates MMP13 as a key regulator in osteolytic bone metastasis [17], where its expression may be induced in osteoblats by tumor-cell derived factors such as oncostatin M and the acute response apolipoprotein SAA3 [18]. It is uniquely expressed by osteoblasts and hypertropic chondrocytes during foetal bone development, but otherwise its expression is minimal under physiological conditions in adult tissues. It is expressed during collagenous tissue repair or remodeling [9], [19], and during fibrogenesis and wound healing, where it has been found to accelerate repair [20], [21]. MMP13 has been implicated in the cartilage damage of human osteoarthritis and rheumatoid arthritis [22], [23] and lack of MMP13 was shown to halt cartilage erosion in established osteoarthritis [24].

MMP13 lends itself to specific inhibition due to a relatively deep S1′ pocket [25]. Computational modeling has led to the identification of a panel of pyrimidinetrione-based inhibitors that are selective for MMP13 due to their binding in its deep S1′ pocket [26], [27]. The inhibitor used in this study, 5-(4-{4-[4-(4-fluorophenyl)-1,3-oxazol-2-yl]phenoxy}phenoxy)-5-(2-methoxyethyl)pyrimidine-2,4,6 (1H,3H, 5H)-trione (Cmpd-1 which is Cmpd-28 in ref. 26), was chosen from a panel of small molecule MMP13-selective inhibitors for its potency and lack of deleterious side effects of a closely related compound in an animal model of MSS [27]. Here we investigate the inhibitory effects of Cmpd-1 on the growth of primary tumors, the incidence and development of tumor-associated osteolytic lesions in the MDA-MB-231 human xenograft and 4T1.2 murine syngeneic models of breast cancer. Our results demonstrate reduced primary tumor growth in both models and delayed development of osteolytic bone lesions in the xenograft model.

## Materials and Methods

### Breast cancer cells

We used human MDA-MB-231 cells transfected with the bacterial β-galactosidase (MDA-MB-231-BAG1 cells) and 4T1.2 mouse mammary cells [28], [29], [30] engineered to express luciferase (kindly provided by A/Prof. Robin Anderson, Peter MacCallum Cancer Centre, Melbourne, Australia; Luciferase expression in 4T1.2 cells by Dr. John Price, Monash Medical Centre, Melbourne, Australia). MDA-MB-231-BAG1 (hereafter referred to as MDA-MB-231) and 4T1.2-Luc (referred to as 4T1.2) cells were routinely cultured in RPMI-1640 and DMEM complete growth medium, respectively (Gibco BRL, Australia) supplemented with 10% fetal bovine serum (Gibco BRL, Victoria, Australia) in a 37°C/5% CO_2_ humidified incubator.

### Induction of primary tumor growth and bone osteolysis by inoculation of breast cancer cells into mice

For the prevention study in the xenograft model, intra-cardiac inoculation of MDA-MB-231 cells into 4 week old female BALB/c *Nu/Nu* mice (Animal Resources Centre, Perth, Australia) was performed as previously described [31], [32]. Briefly, each mouse was inoculated under anesthesia in the left ventricle with 2×10^5^ MDA-MB-231 cells in 100 µl PBS. Another group of 4 week old female mice were inoculated with MDA-MB-231 cells bilaterally into the mammary fat pad (5×10^5^ cells in 15 µl PBS into each), as previously described [31]. 4T1.2 cells were inoculated bilaterally into the mammary fat pad (5×10^5^ cells in 15 µl PBS into each site) of 5–7 weeks old female wild-type BALB/c mice (Animal Resources Centre) for the prevention study as described above. For the intervention study, BALB/c *Nu/Nu* female mice were inoculated with MDA-MB-231 breast cancer cells in the mammary fat pad, as described above, with treatments started later as detailed below. The mouse studies were conducted with approval of the St Vincent's Hospital Animal Ethics Committee (AEC#35/08) and in accordance with the Australian National Health and Medical Research Council's guidelines for the care and use of laboratory animals.

### Administration of Cmpd-1

**Figure 1:**
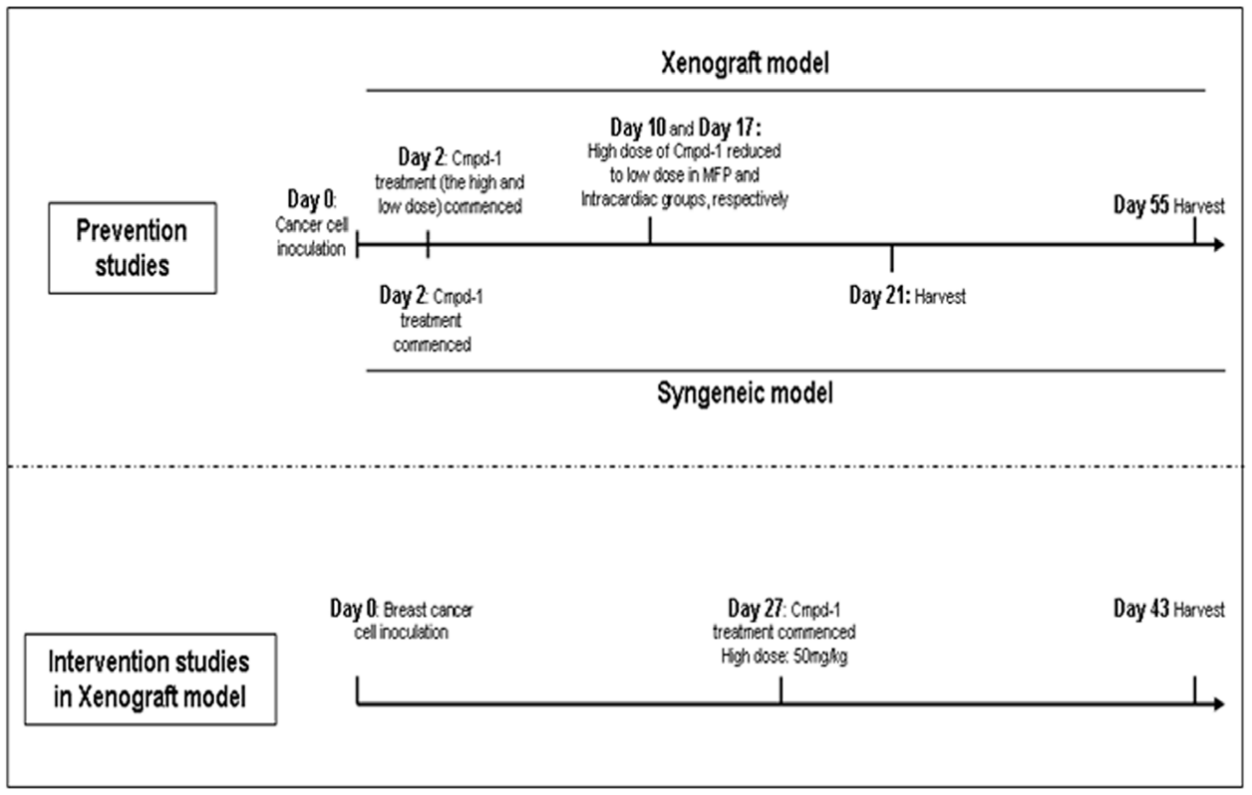
Cmpd-1 treatment timeline of prevention and intervention studies in xenograft and syngeneic mouse models.

Cmpd-1 powder was synthesized at Pfizer (Groton, CT, USA) and prepared freshly in vehicle solution (20 mM citric acid, 3.5 mM sodium citrate, 50% v/v polyethylene glycol 400, 10% w/v hypromellose (Sigma-Aldrich). A schematic presentation of the timeline for Cmpd-1 treatment used in the various mouse studies is shown in Figure 1. For the prevention studies, the mice were dosed from 2 days after the inoculation of the cells by oral gavage with vehicle (n = 10) or Cmpd-1 at low dose (25 mg/kg of mice body weight; n = 10) or high dose (50 mg/kg; n = 10), twice daily at ∼12 h intervals. These doses were chosen to provide selective inhibition of MMP13 over other MMPs (see discussion) and had been well tolerated by rats in previous Pfizer studies (data not shown). However, due to some adverse observations, the high dose regimen was reduced to low dose on Days 10 and 17 for the MDA-MB-231 mammary fat pad and intra-cardiac experiments, respectively. The high dose regimen was therefore renamed as the two-tier regimen for the MDA-MB-231 experiments. These adverse responses were not seen in later experiments. An additional group of intra-cardiac inoculated mice (n = 10) commenced weekly dosing with the bisphosphonate zolendronic acid (Zometa; supplied by Novartis, Basel, Switzerland - sc injections of 100 µg/kg) for three weeks based on previous studies [33], with the first dose provided 3 days after cancer cell inoculation. The 4T1.2-inoculated BALB/c mice were dosed with either vehicle (n = 8) or Cmpd-1 at low dose (25 mg/kg of mice body weight; n = 8) or high dose (50 mg/kg; n = 8) by oral gavage twice daily at ∼12 h intervals, starting on Day 2. The high dose of Cmpd-1 was continued throughout the experiment duration until the harvest. An intervention study was performed with the MDA-MB-231 xenografts to examine the effect of Cmpd-1 on established tumors and mimic the treatment in human breast cancer patients. The mice were monitored for tumor growth after mammary fat pad inoculations of MDA-MB-231 cells and randomised for the Cmpd-1 treatment when the average tumor sizes were ∼120 mm^3^. The mice were dosed from Day 27 with either vehicle (n = 7) or high dose Cmpd-1 (50 mg/kg of mice body weight; n = 8) by oral gavage twice daily at ∼12 h intervals, until the end of the experiment on Day 43.

### X-ray analysis, tumor measurements, bioluminescent imaging and harvesting of tumors, blood and soft organs

For the mammary fat pad xenograft model, tumor-bearing mice were monitored thrice weekly for body weight and tumor size. Once palpable, the tumor sizes were measured thrice weekly in two dimensions using digital calipers, and culled when tumor size approached 10% of body weight. To assess osteolytic damage of intra-cardially inoculated MDA-MB-231 mice, the hind limbs were subjected to high resolution X-ray analysis (Faxitron, USA; 20 µm focal source) thrice a week from week 2, as previously described [32]. These mice demonstrated extensive bone destruction and were harvested as soon as they showed any adverse signs or symptoms of disease or morbidity; otherwise at an experimental endpoint of 55 days post inoculation. Blood collected immediately prior to organ harvest was obtained by cardiac puncture from a limited number of mice and allowed to clot overnight at 4°C, after which the serum was separated by centrifugation at 400 g and stored at −80°C until analysis. Lungs and brain were snap-frozen and the level of metastatic burden quantitated by Alu PCR, as described below.

The mice bearing 4T1.2 cells, in addition to the routine monitoring as described above, were subjected to luminescence detection using the IVIS system (Xenogen). Mice were anesthetized as described previously [31], [32], and luciferase signal was measured by intraperitoneal delivery of luciferin (112 mg/kg in PBS; 15 mg/ml) 10 mins before imaging and analysing photon emissions from the tumor cells at fixed time points over the length of the experiment. Ex-vivo lung imaging was performed immediately following the euthanising of mice, to detect the amount of lung metastasis. These mice were harvested after 3 weeks of cell inoculations unless harvested early due to signs of distress. Luminescence intensity was used to quantify the metastasis in lung.

### Quantification of metastatic burden in soft tissue organs

 Alu PCR analysis was performed to quantitate metastatic burden in lungs and brain collected from mice inoculated intracardially with MDA-MB-231 cells, as previously described [34], on 10 ng of purified genomic DNA in a Rotor-Gene 6000 series system (Corbett Research, Sydney, Australia). On each 72-well reaction disk, a standard curve was prepared by serially diluting human DNA into mouse DNA, permitting the quantification of the tissue burden of human tumor cells in the mouse organs removed at autopsy. Metastatic burden is indicated by the proportion of human DNA found in each organ, expressed as a percentage.

In the 4T1.2 model, representative lungs (n = 4 per group) were fixed in formalin, embedded in paraffin and subjected to serial sections of 10 µm thickness. The process was repeated through the entire lung and every tenth section was stained with Haematoxylin and Eosin following routine procedures.

### Analysis of serum concentrations of Cmpd-1 and creatine kinase

Cmpd-1 concentrations in serum were quantified using a high performance liquid chromatography–tandem mass spectrometry (HPLC/MS/MS) assay. Serum samples were thawed on ice and 150 µl of acetonitrile containing 5-(4-{[1-(4-fluorophenyl)-1H-indazol-5-yl]oxy}phenoxy)-5-(2-methoxyethyl)pyrimidine-2,4,6(1H,3H,5H)-trione (an analogue of Cmpd-1) was added as an internal standard to 50 µl of undiluted sample, diluted sample (1 part serum and 9 parts control serum), and standard. The samples were then vortexed and centrifuged at 500 g for 10 minutes. The supernatants (150 µl) were transferred to a 96-well plate for analysis by HPLC/MS/MS, using transition state monitoring of 532.2/347.2 and 505.5/320.2 for Cmpd-1 and its analogue, respectively, in the positive ion mode. The dynamic range of the assay was 0.010 to 10 µg/ml.

As a general measure of musculoskeletal toxicity, which has been reported for MMP inhibitors, we assessed serum levels of creatine kinase collected from the 4T1.2 experiment following the manufacturer's protocol (CK-NAC, Beckman Coulter Inc. CA, USA).

### Statistical analyses

Data were analysed using Graph Pad Prism 5.0 (GraphPad Software Inc). Two-way repeated measures (RM) ANOVA was performed to compare the mammary fat pad tumor sizes between control and treatment groups. The Bonferroni post-test was performed to determine at which time points tumor sizes varied between treatment regimens. Tumor did not grow in one of the mammary fat pads of a mouse in the vehicle control group for MDA-MB-231, and this sample was excluded from the analysis, as was the data from two mice in the low Cmpd-1 group that died early in the experiment due to unrelated health issues. Time to reach specified lesion scores for osteolytic damage between control and treatment groups and percent survival after cancer cell inoculation were analysed using the Kaplan-Meier method, with P values calculated using the log-rank test. Metastasis levels to soft organs determined by Alu PCR were compared using the Mann-Whitney test. One-way ANOVA was performed to compare serum levels of CK between the control and treatment groups. Values are indicated as mean ± SEM. P values<0.05 were considered statistically significant in all comparisons.

### MMP13 immunofluorescence and localisation in primary breast tumors and bone metastasis clinical samples, and in experimental tumors

Human breast tumors were collected with the approval of the St Vincent's Hospital Human Research Ethics committee (SVH HREC-A), Melbourne, Australia. The need for individual patient consent was waived by the SVH HREC-A as tissues were collected by the hospital for hospital procedures and individual patients cannot be identified unduly, and the project would not impact on tissue donors' disease or treatment. The clinico-pathological characteristics and formalin-fixed paraffin-embedded (FFPE) tumor sections of primary breast invasive ductal carcinomas (IDCs) from patients (n = 8) who underwent surgery and had no preoperative treatments, were obtained from the archives of the Dept. of Anatomical Pathology, St. Vincent's Hospital, Melbourne. Immunofluorescence on tumors from clinical breast cancer samples, and xenograft and syngeneic mouse models was performed essentially as described [11], using sheep anti-human MMP13 serum (kindly provided by Prof. Gillian Murphy, Cambridge Institute of Medical Research, UK). Fluorescence microscopy was performed on an AxioCam MRC5 instrument and images were acquired at 40× magnification using Axiovision v4.8 software (Zeiss, Göttingen, Germany). Propidium iodide was used as a nuclear stain.

Human de-identified samples of frank breast to bone metastases (n = 5) were obtained under an Institutional Review Board approved protocol at Vanderbilt University Medical Center, Nashville, TN, USA. All human specimens for research were collected with patients' written consent. Fluorescent tartrate-resistant acid phosphatase (TRAcP) staining for osteoclast detection (Elf97; Invitrogen) and immunofluorescent staining for MMP13 (1∶250 dilution; RP1-MMP13, Triple Point Biologics, USA) was performed from FFPE sections as previously described [35], [36]. Rabbit IgG (Sigma-Aldrich) was used as a control but neither staining for the isotype control nor autofluorescence in the red channel (ex-568 nm) was detected (data not shown). DAPI, at a final concentration of 1 nm was used as a nuclear stain.

### Ki67 staining of MDA-MB-231 Bag1 and 4T1.2-Luc primary tumors

IHC analysis was performed for Ki67 on 5 µm FFPE sections from xenograft and syngeneic mice tumors. To retrieve the antigenicity, the tissue sections were boiled in 10 mmol/L citrate buffer (pH 6.0) for 20 min. IHC was performed using the peroxidase method. The primary antibodies used were mouse anti-human Ki67 (Clone MIB1 #3707: DAKO Australia @ 1∶100) and rabbit anti-mouse Ki67 (clone SP6 #RM-9106-S0; Labvision Australia @ 1∶500). Non-immunized IgG (DAKO Australia) at an equivalent concentration to the primary target antibody was used as a negative control. Endogenous peroxidases were blocked with 3% H_2_O_2_. Slides were incubated with primary antibodies at 4°C overnight followed by the incubation with secondary antibody (Dako rabbit anti-mouse and goat anti-rabbit HRP secondary at 1∶1000 dilution) for 1 hour at room temperature before visualisation with DAB for 8 min. Slides were counterstained with Mayer's haematoxylin.

## Results

### Early treatment of Cmpd-1 impacts upon the growth of primary tumors and the onset and severity of osteolytic lesions in the xenograft model: a prevention study

**Figure 2:**
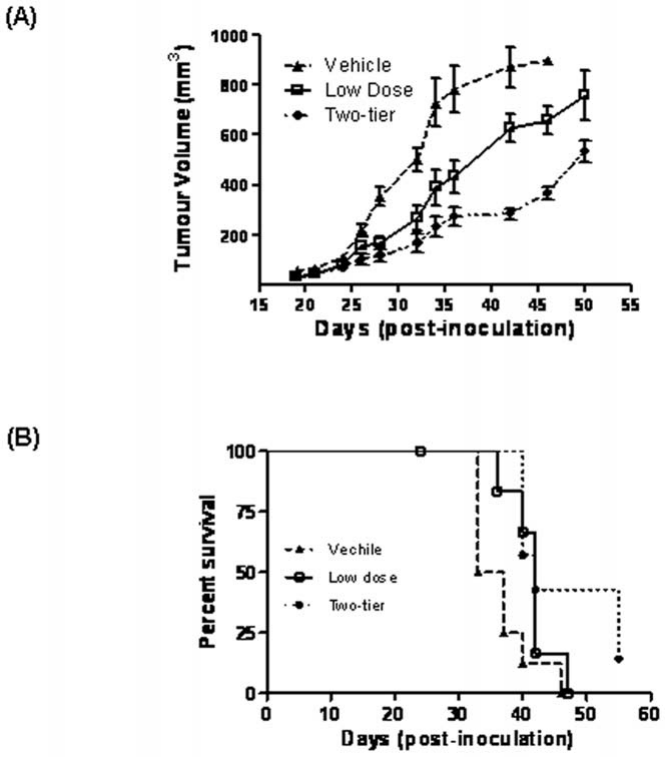
Prevention studies with Cmpd-1 and its effect on primary tumor growth in the MDA-MB-231 human breast cancer xenograft model. (A) Three groups of mice inoculated with cells at day 0 were treated with vehicle (n = 10) or either of the low dose (25 mg/kg twice daily; n = 10) or two-tier (50 mg/kg for initial 10 days and then reduced to low dose; n = 10) Cmpd-1 regimens from day 2 until the end point of the experiment. There was a significant dose-dependent effect of Cmpd-1 on the inhibition of primary tumor growth (vehicle *vs.* low dose p = 0.0018; vehicle *vs.* two-tier p<0.0001; ANOVA and Bonferroni post-test). Tumor measurements plotted are expressed as the mean ± S.E.M. (B) Mice treated with low or two-tier Cmpd-1 regimen showed prolonged survival compared to vehicle control (p = 0.0066 and p<0.0001, respectively; Kaplan-Meier survival).

Mouse body weights did not differ between Cmpd-1 treatment groups over the course of the experiment (data not shown). Early deaths seen in the low Cmpd-1 regimen MDA-MB-231 mammary fat pad group appeared unrelated to Cmpd-1 since they were not seen in the high dose group, as also confirmed in the following experiments. As demonstrated in Figure 2A, primary MDA-MB-231 xenograft growth was markedly inhibited by treatment with either the low Cmpd-1 regimen (p = 0.0018) or the two-tier Cmpd-1 regimen (p<0.0001), the latter being more effective than the former (p = 0.043). Differences in tumor size between vehicle control and treatment groups became significant from 28 days post inoculation (Figure 2A). Furthermore, Kaplan-Meier survival in mice treated with low (p = 0.0066) or two-tier (p<0.0001) Cmpd-1 was prolonged compared to vehicle control, as determined at the time of harvest (Figure 2B).

**Figure 3:**
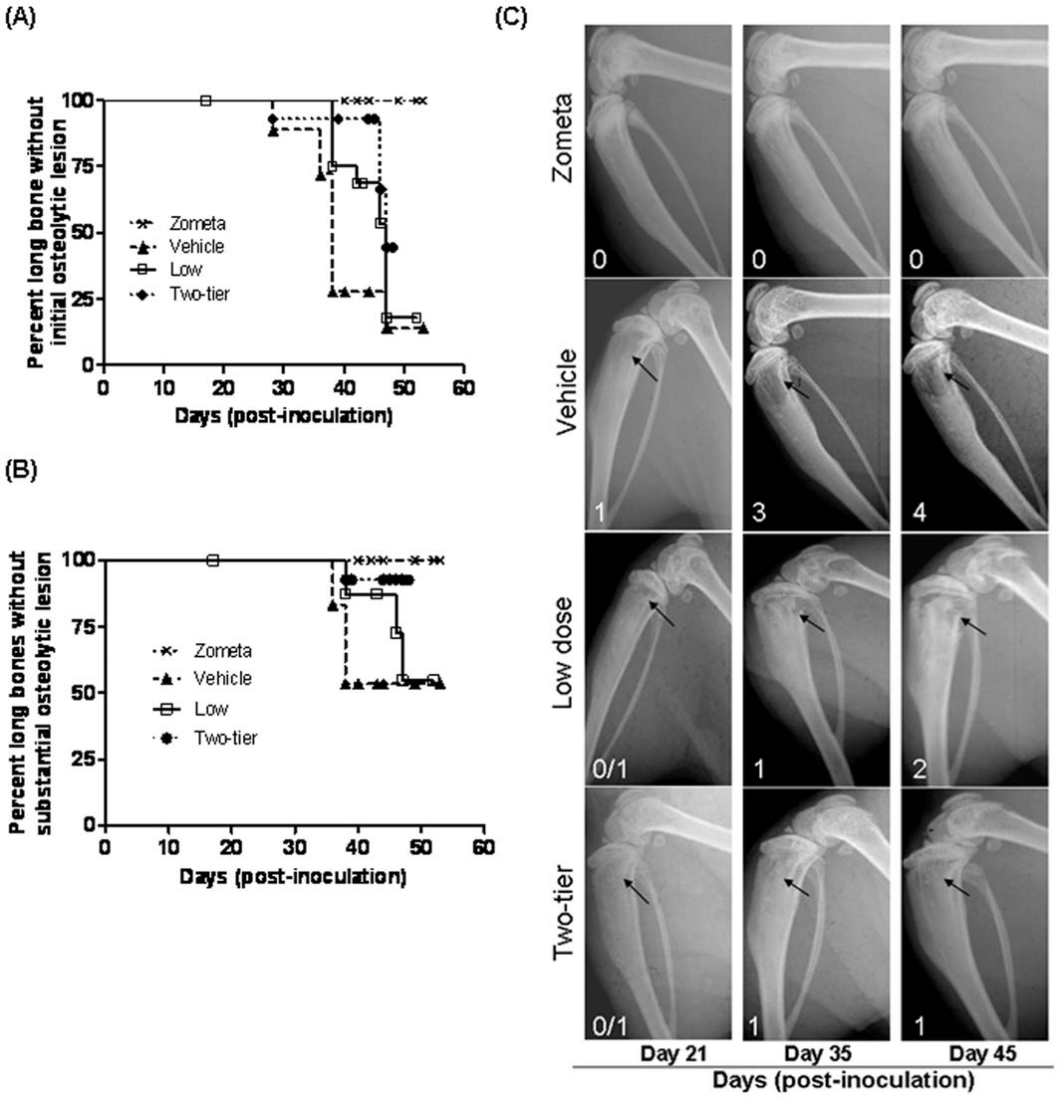
Prevention treatment with Cmpd-1 and its effect on osteolytic damage in the tibiae bones. (A) Three groups of mice were inoculated with MDA-MB-231 cells intracardially at day 0 and were treated with vehicle (n = 10) or either of the low dose (25 mg/kg twice daily; n = 10) or two-tier (50 mg/kg for initial 17 days and then reduced to low dose; n = 10) Cmpd-1 regimens from day 2 until the end point of the experiment. A fourth group of mice (n = 10) received weekly treatment of Zometa for 3 weeks beginning on day 3. The long bones were analyzed by high resolution X-ray analysis and the degree of osteolytic damage was scored on a semi-quantitative scale, with a score of 0 representing no visible damage and a score of 4 representing severe damage. The percentage of mice without (A) initial osteolytic damage (Cmpd-1 low p = 0.027, two-tier p = 0.0037) or (B) substantial osteolytic damage (Cmpd-1 low p = NS, two-tier dose p = 0.017) of the proximal tibia was significantly lower when treated with Cmpd-1 compared to vehicle-treated mice (Kaplan-Meier analysis). (C) Examples of osteolytic lesions (shown using black arrowheads) in long bones following the treatment with vehicle alone, Zometa or Cmpd-1 given under either the low dose or two-tier regimens are illustrated in the panel and the lesion score is displayed on respective image.

The degree of osteolytic damage in the proximal tibia of mice following intra-cardiac seeding of MDA-MB-231 cells was scored using a semi-quantitative scale (where a score of 0 represents no visible osteolytic damage, 1 represents visible damage that may disappear in the subsequent analysis, 2 represents definite damage unlikely to disappear, 3 represents substantial damage and 4 represents severe/extensive damage) [32]. The times to reach the onset of definite osteolytic damage (score 2, Figure 3A) and substantial osteolytic damage (score 3, Figure 3B) were analyzed. Zometa, a bisphosphonate known to block osteoclast mediated bone resorption in mouse models of breast-bone metastasis, completely abrogated the formation of osteolytic bone lesions while both treatment regimens for Cmpd-1 caused a significant reduction in lesion onset shown in Figure 3A (score 2, vehicle *vs* low Cmpd-1 p = 0.027, vehicle *vs* two-tier Cmpd-1 p = 0.0037). A significant reduction in the incidence of substantial osteolytic damage was also seen in mice treated with two-tier Cmpd-1 (score 3, vehicle *vs* two-tier Cmpd-1 p = 0.017; Figure 3B), but not low Cmpd-1. The inhibitory effects of low and two-tier Cmpd-1 are shown in the representative Faxitron images of Figure 3C.

### Cmpd-1 delays the primary tumor growth in a syngeneic murine model of breast cancer

**Figure 4:**
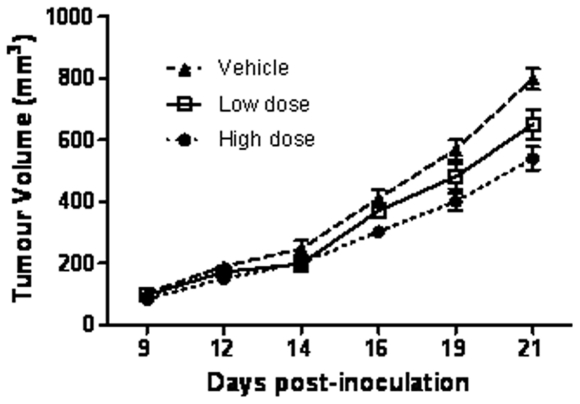
Prevention treatment with Cmpd-1 and its effect on primary tumor growth in a syngeneic mouse model of breast cancer. Three groups of mice were inoculated with 4T1.2 cells into mammary fat pad at day 0 and were treated with vehicle (n = 8) or either of the low (25 mg/kg twice daily; n = 8) or high (50 mg/kg; n = 8) Cmpd-1 regimens from day 2 until the end point of the experiment. The primary tumor growth was significantly delayed in mice treated with low or high dose of Cmpd-1 compared with vehicle-treated mice (p = 0.0216 and p<0.0001, respectively; RM-ANOVA with Bonferroni post-test). Each time point represents tumor measurements as mean ± S.E.M.

Mammary fat pad growth of 4T1.2 mouse mammary tumor cells was markedly inhibited in mice receiving either the low or the high Cmpd-1 regimen (p<0.0003) compared to the vehicle group. The high dose regimen was more effective from earlier time points (day 16, 19 and 21, p≤0.05, <0.001 and <0.001, respectively; Bonferroni post-test) than the low dose regimen (only at day 21 p<0.05; Bonferroni post-test). Differences in tumor size between vehicle control and treatment groups were not significant before 14 days post inoculation (Figure 4).

### Cmpd-1 does not affect metastatic burden in soft tissues

**Figure 5:**
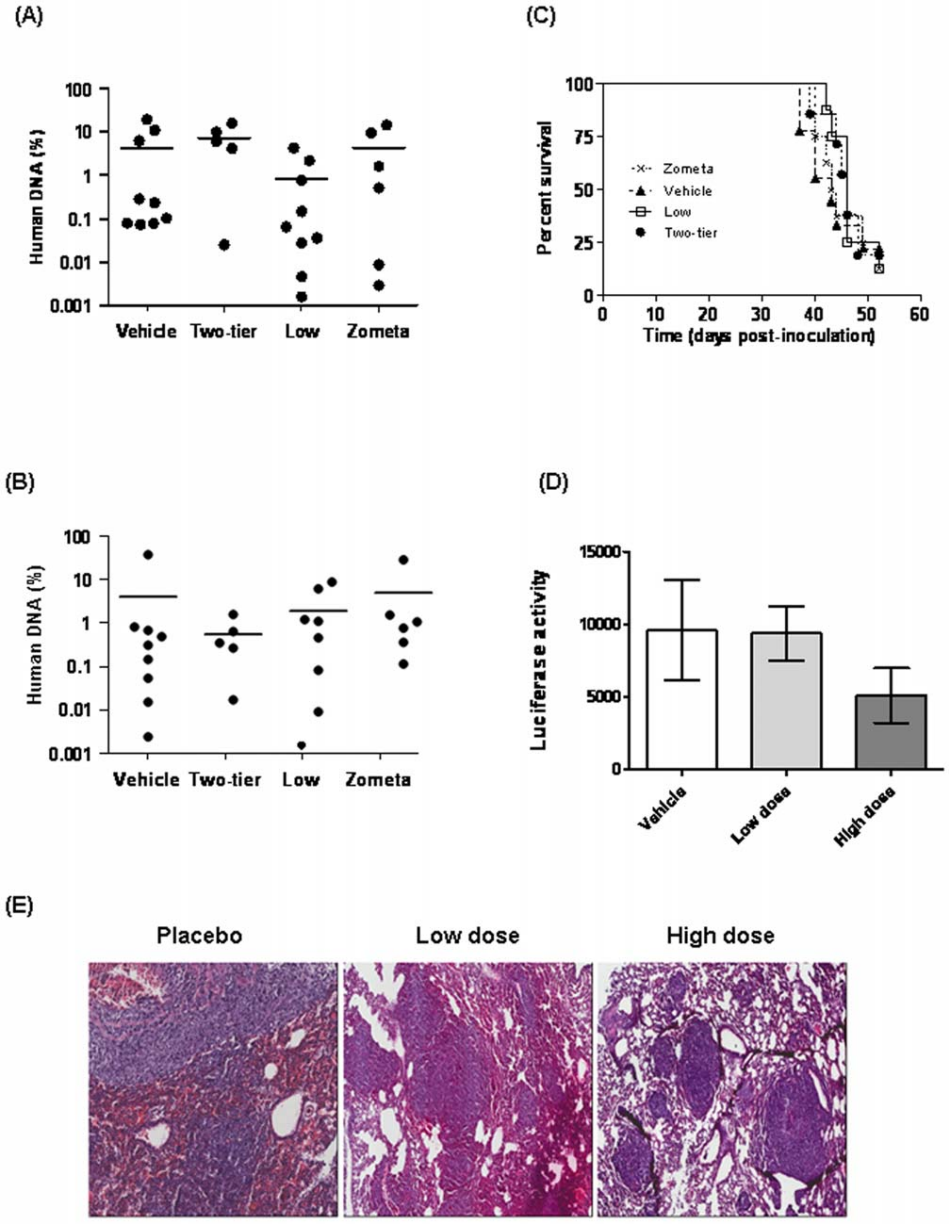
Metastatic burden in the lungs (A) and brain (B) of mice treated with vehicle or Cmpd-1 (low or two-tier regimens) following intra-cardiac inoculation of MDA-MB-231 cells, as assessed by qPCR for the human Alu repeat sequence on DNA extracted from whole organs. Mouse only controls were included in the qPCR analysis and all mouse only samples were negative for human DNA. Mean values are indicated. (C) Percentage survival of intra-cardially inoculated mice. Cmpd-1 did not prolong overall survival of the mice treated with Cmpd-1 (Kaplan-Meier survival). (D) Metastatic burden in the lungs of mice treated with vehicle or Cmpd-1 (low or two-tier regimens) following mammary fat pad inoculation of spontaneously metastasizing 4T1.2 cells. Tumor burden in lungs was assessed by luminescence intensity of *ex-vivo* lung imaging using IVIS at the termination of the experiment. Cmpd-1 showed no inhibitory effect on metastatic tumor burden on soft tissues in both xenograft and syngeneic mice models (Mann-Whitney test). (E) Representative images of lung macrometastasis in mice inoculated with 4T1.2-Luc and treated placebo/low dose/high dose.

In order to assess whether the metastatic burden in soft tissues was affected by treatment with Cmpd-1, quantitative PCR for the human Alu repeat sequence was performed on genomic DNA isolated from the brains and lungs of the mice inoculated intracardially with MDA-MB-231 cells. The results are presented as the percentage of human DNA within each organ (Figure 5A–B). No effect on metastatic tumor burden was observed with either Cmpd-1 or Zometa, in line with the observation that overall survival, represented by time of sacrifice, was not impacted upon by treatment (Figure 5C).

The spontaneous metastases to the lung from mammary fat pad 4T1.2 tumors was assessed at the termination of the experiment using *ex-vivo* IVIS imaging. As shown in Figure 5D, although the high dose regimen showed a trend towards reduced lung metastases, no significant difference was observed in the degree of lung metastasis in animals receiving low or high Cmpd-1 and the vehicle controls. Histological analysis of the lungs from the mice inoculated with 4T1.2-Luc in mammary fat pad (n = 4 per group) revealed extensive lung metastases in each of the treatment groups (Fig. 5E). Although the lung metastases in the high dose Cmpd-1 treatment group appeared to be smaller, there was no significant difference among the groups based on the metastatic lesion counts from five randomly chosen fields. This is in agreement with the luciferase intensity measured in these lungs (Fig. 5D). The lungs from MDA-MB-231 experiments were sacrificed for Alu analysis, and could not be subjected to this analysis.

### Effect of Cmpd-1 treatment on established tumor growth in the xenograft model: An intervention study

**Figure 6:**
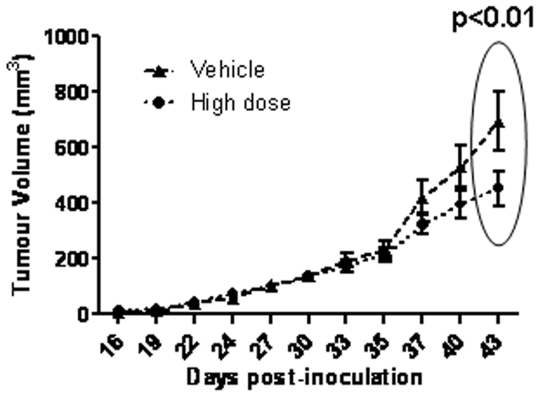
Intervention treatment with Cmpd-1. Mice were inoculated with MDA-MB-231 cells at day 0 and randomized to receive either vehicle (n = 7) or high Cmpd-1 regimen (50 mg/kg; n = 8) from day 27 continued until harvest (day 43). Each time point of tumor measurements plotted on the graph represents the mean ± S.E.M. Cmpd-1 inhibits established tumors in high dose treated mice at the largest tumor size (p<0.01 by student-t test, p = NS by RM-ANOVA).

An intervention study was undertaken with the high dose regimen to test whether the Cmpd-1 was able to inhibit the growth of established MDA-MB-231 xenografts. At day 27, when the average tumor size was ∼120 mm^3^, mice were randomized into two groups receiving either Cmpd-1 or vehicle twice daily at ∼12 h intervals by oral gavage until harvest. At day 35, no significant difference in tumor size was observed between the two groups, thereby demonstrating successful randomization of mice (Figure 6). Cmpd-1 was effective on established tumor growth inhibition at the harvest time point (day 43) (p<0.01), but did not achieve significance over the entire treatment period by RM-ANOVA.

### Serum concentrations of Cmpd-1 are well maintained, and creatine kinase is not elevated

**Figure 7:**
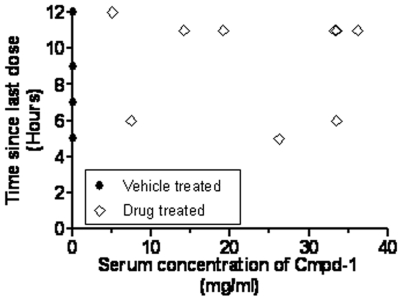
Concentration of Cmpd-1 in serum collected at the time of culling plotted against the time since last dose of Cmpd-1.

Blood was collected from a number of the intra-cardiac inoculated mice at sacrifice and the serum concentration of Cmpd-1 measured to determine whether it was concordant with previous pharmacokinetic data (Pfizer, unpublished). For the samples assessed, the serum concentration of Cmpd-1 was in the range of 5–36 µg/ml (Figure 7). Although the mice were sacrificed at different time points following their final dose of Cmpd-1 (5 to 6 hours or 11 to 12 hours), the distribution of Cmpd-1 serum levels was similar.

The serum levels of creatine kinase from the 4T1.2 experiment showed no significant differences (p = 0.1339; one-way ANOVA) when compared the vehicle control group (n = 7, 280.7±127.7) with low Cmpd-1 (n = 7, 537.2±222.1) or high Cmpd-1 (n = 5, 210.0±140.6) (data not shown).

### MMP13 is present in the intratumoral and extracellular/tumor-stromal regions and vascular endothelial cells of clinical primary breast cancer and experimental mice tumors, and in osteoblasts in the breast to bone metastases

Since we observed inhibition of primary tumor growth and osteolytic lesions with Cmpd-1 treatment in mouse models, we further examined MMP13 localization in clinical samples.

**Figure 8:**
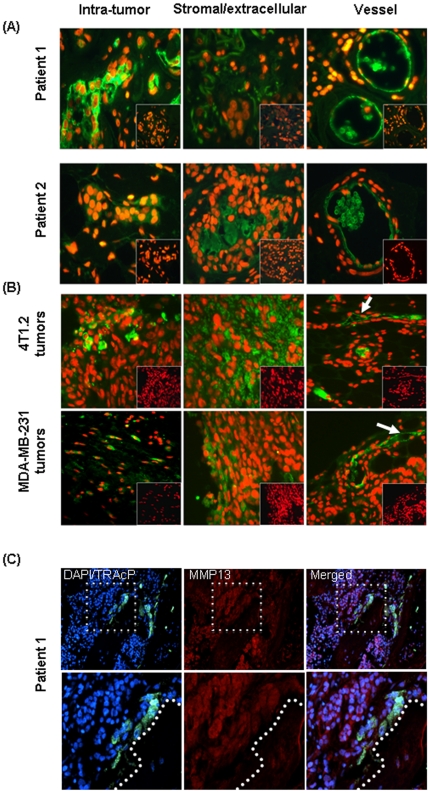
Immunofluorece for MMP13. (**A**) MMP13 immunofluorescence images of the clinical breast tumors from Patients 1 and 2. Immunofluorescence staining was performed on FFPE sections for MMP13 using a sheep polyclonal MMP-13 antibody as indicated by green fluorescence, whereas nuclei are stained red with propidium iodide. Parallel sections from the same tissue blocks were probed with control sheep serum to monitor for non-immune background (lack of green signal displayed as an inset in each panel). (**B**) Representative images of MMP13 immunofluorescence (green) from the syngeneic (top panel) and xenograft (bottom panel) mouse tumors. (**C**) MMP13 immunofluorescence (red) in human breast to bone metastasis. Dashed box in upper panels represents area of magnification in lower panels. Multinucleated (DAPI; blue) osteoclasts (TRAcP; green) were identified at the tumor-bone interface (represented by dashed line in the lower panels).

Primary human breast IDC samples (n = 8) and breast-to-bone metastases (n = 5) were stained by immunofluorescence. The proportion of cancer cells expressing MMP13 varied greatly, ranging from no detectable MMP13 in 25% of patient samples, through focal expression, to expression throughout most of the tumor. Focal expression of MMP13 was also detected within tumor-associated stroma or extracellular matrix in 75% of patient samples, and all patient samples showed strong MMP13 immunoreactivity in the vasculature. The immunoreactivity of MMP13 in primary tumors is summarised in Table 1, and examples of MMP13 for two representative patients are shown in Figure 8A. The immunolocalisation of MMP13 has also been confirmed in the experimental mice tumors from xenograft and syngeneic models using the above validated method. As depicted in Figure 8B, in each case intratumoral staining, more abundant stromal/extracellular staining, and prominent vascular staining was observed.

**Table 1:** Clinicopathologic details of breast cancer patients.

Patient #	Lymph node Metastasis	Histological Grade (1–3)	Estrogen receptor Status	Progesteron receptor Status	MMP13 Expression
1	Yes	2	+++	+++	T,S,V
2	No	1	+++	−	T,S,V
3	N/A	1	+++	+++	T,V
4	No	1	+++	+++	T,V
5	No	2	++	++	T,S,V
6	N/A	2	+++	+++	S,V
7	Yes	2	+++	+++	T,S,V
8	Yes	2	+++	−	S,V

Summary of MMP13 expression and clinicopathologic characteristics of primary breast tumors.

Tissue samples were graded and MMP13 expression was determined by immunofluorescence, as described in Materials and Methods. All patients were diagnosed with invasive/infiltrating ductal carcinoma (IDC). The MMP13 immunofluorescence images of the samples from Patients 1 and 2 are shown in Figure 7. N/A = not available. The tumors from all the patients, except patient 7 where HER-2 status was not determined, were negative for HER-2. Estrogen receptor and progesterone receptor status was graded as − = negative, + = weak, ++ = intermediate and +++ = strong expression. MMP13 expression was determined as intra-tumor (T), stromal (S) and vasculature (V).

The human bone metastases samples revealed positive MMP13 [33] staining by the tumor cells and osteoblasts cells while multinucleated (blue) TRAcP positive osteoclasts (green) appeared largely negative (Figure 8C).

### Proliferation remained unchanged in Cmpd-1 treated vs untreated mice tumors

**Figure 9:**
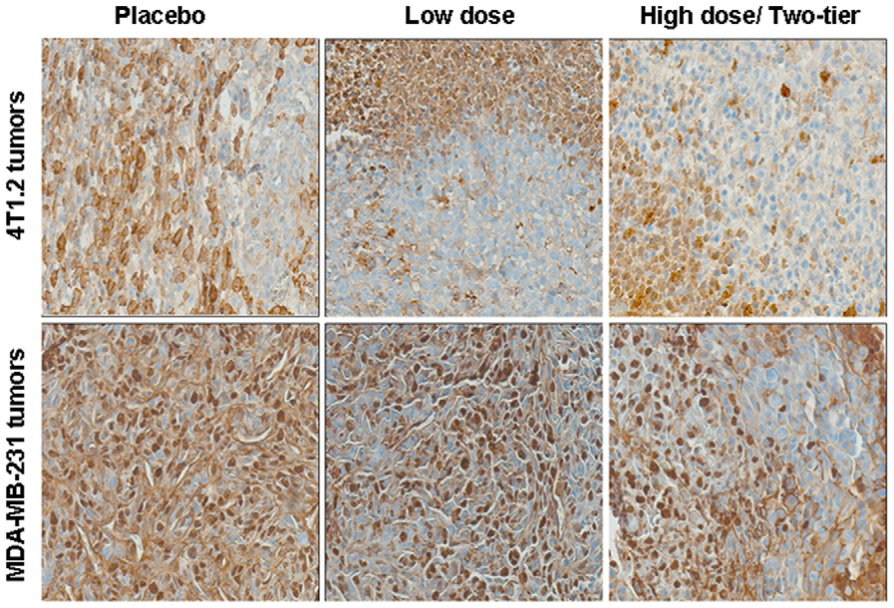
Expression of Ki67 in the experimental mice tumors from syngeneic (top panel) and xenograft (bottom panel) models treated with either vehicle, low and high dose of Cmpd-1.

To examine the effect of Cmpd-1 on tumor cell proliferation, we assessed the experimental mice tumors from each treatment groups using Ki67 immunohistochemistry. As shown in representative images (Fig. 9), we observed abundant Ki67 positive nuclear staining across all tumors from each treatment groups, with no marked difference in the proliferation index.

## Discussion

Cmpd-1 is an MMP13-selective inhibitor chosen from a panel of pyrimidinetrione based molecules [27]. Its selectivity is high for MMP13 (IC50 of 0.57 nM MMP13, 950 nM MMP1, 370 nM MMP8, 200 nM MMP2, 360 nM MMP9, 7900 nM MMP3, and 550 nM MMP12) [27]. It is anticipated that such MMP-selective or -specific inhibitors will provide better therapeutic outcomes, overcoming some of the problems encountered with previous broad-spectrum inhibitors. Whilst broad-spectrum MMP inhibitors were impressive in animal studies, many of these drugs did not prove effective in the clinical setting, where a number of trials showed toxicity such as MSS and also a lack of efficacy [37], [38]. MSS seems not attributable to MMP13, as an orally available MMP13 specific inhibitor developed through structure-based drug design did not induce MSS symptoms in a rat model when used to treat cartilage damage [39]. In the present study, serum levels of creatine kinase, which is a general measure of muscle toxicity, remained unchanged in mice treated with either low or high dose of Cmpd-1 when compared with untreated group.

Our studies appear to have achieved pharmacologically relevant dosing throughout the duration of Cmpd-1 administration. Eleven to 12 hours after final drug administration the serum levels of Cmpd-1 ranged from 5 to 36 µg/ml and were similar in samples taken 5 to 6 hours after final dosage. These findings are in line with previous pharmacokinetic profiling, where a maximal plasma concentration of 16 µg/ml was obtained 4 hours after a single oral administration of Cmpd-1 (25 mg/kg), dropping to 4 µg/ml by 24 hours (unpublished data). As calculated from the unbound fraction of Cmpd-1 in mouse plasma (0.62%; unpublished data), the lowest serum concentration of unbound Cmpd-1 observed in our study equates to approximately 110-fold and 20-fold the MMP13 IC_50_ for human (0.57 nM) [27] and rat (3.2 nM; unpublished data) respectively. The rat MMP13 IC_50_ is likely to be a reasonable estimate of the mouse MMP13 IC_50_, as the sequence of the secondary structure elements of the rat and mouse MMP13 active sites are identical [40].

We have shown that treatment with the MMP13-selective inhibitor Cmpd-1 reduced the growth rate of two different primary breast cancer models: The MDA-MB-231 human breast cancer xenograft and the 4T1.2 syngeneic mouse model. Cmpd-1 was effective as a preventative agent in both models, and as an interventional agent in the MDA-MB-231 xenograft model, although the intervention effect was only apparent at largest tumor sizes. Previously, broad-spectrum MMP inhibitors have shown little benefit against pre-established preclinical models [41] (also our unpublished data with AG3340 in the MDA-MB-231 model), so this effect is particularly important in light of the low efficacy in previous clinical trials. During the latter stages of the experiment the two-tier Cmpd-1 regimen in xenograft mice exhibited a larger reduction in primary tumor growth rate than the low Cmpd-1 regimen mice, despite both treatment groups receiving the same low dose Cmpd-1 regimen from day 10 onwards. Similarly, a dose-dependent effect of Cmpd-1 was observed on primary tumor growth in the syngeneic model of mammary cancer. The latency period of these tumors did not differ with treatment. Further, tumor cell proliferation with and without Cmpd-1 treatment was evaluated. No obvious difference in Ki67 positive staining was demonstrated among the groups from xenograft and syngeneic models. These observations suggest that the Cmpd-1-associated reduction in the primary tumor growth rate may involve number of different factors that may or may not relate to remodeling of the extracellular, and/or vascularisation factors, and is the subject of further investigation. Recently, Overall and coworkers identified a number of novel MMP13 cleavage substrates that may be involved, including inactivation of the chemokine CCL2, activation of platelet derived growth factor-C and cleavage of SAA3, osteoprotegrin, Cut A and antithrombin III [18].We speculate that early treatment of Cmpd-1 may have reduced angiogenic seeding in the initial primary tumor mass, but further analysis is required.

Primary tumors showed immunoreactivity of MMP13 in the tumor cells and surrounding stroma/extracellular matrix. Previously we have shown that MMP13, while barely detectable in naïve mammary fat pads, was abundantly expressed in the tumor-associated murine stroma of breast cancer cell line and primary tumor xenografts [11]. Such MMP13 localisation in the xenografts was confirmed and extended to the mouse syngeneic model in the present study. A study of 263 cases of invasive breast cancer revealed MMP13 expression by both tumor cells and adjacent fibroblasts, with high levels of MMP13 in these two cell types strongly correlated with each other [5]. MMP13 immunoreactivity in the cytoplasm of tumor cells has also been identified in 91% of 249 colorectal cancers resections [8]. A previous *in situ* hybridisation study in ductal carcinoma *in situ* (DCIS) lesions reported only stromal MMP13 mRNA expression and identified the MMP13 positive cells as being myofibroblasts. Expression of MMP13 was correlated with microinvasion events, leading the authors to postulate that MMP13 may play a role in the transition of DCIS to IDC [42]. Prominent MMP13 staining in the tumor vasculature of IDC and in the mouse tumors of the present study warrants further assessment. Recent studies support involvement of MMP13 in tumor angiogenesis during melanoma progression [10] and skin carcinoma [43].

Another important observation of this study was that Cmpd-1 treatment delayed the onset and reduced the severity of tumor-induced osteolytic lesions in the experimental xenograft model of bone metastasis following intra-cardiac inoculation of breast cancer cells. A reduction in the incidence of substantial bone lesions was observed for the two-tier Cmpd-1 regimen group, but not the low dose regimen mice, despite both groups receiving the same dosage from day 17 onwards. This change to lower dose was prior to the first appearance of osteolytic damage. The anti-osteolytic effects of Cmpd-1 treatment may result from reduced tumor growth in the bone environment, similar to the reduced primary tumor growth rate, and/or from direct inhibition of osteolytic pathways. The present findings are further supported by Nannuru et al, who found that treatment with MMP13 antisense oligonucleotides reduces mammary tumor-induced osteolysis at the tumor-bone interface, where they also found elevated MMP13 [17]. Furthermore, it has been shown that mice lacking MMP13 demonstrate delayed growth plate formation in the long bones [12], [24], [44], and this is much more prominent in mice lacking both MMP9 and MMP13 [13]. Thus, MMP13 inhibition may affect both the growth and osteolytic consequences of breast carcinoma cells metastasized to bone. These inhibitory effects of Cmpd-1 (MMP13-selective inhibitor) are consistent with the effects of several broader-spectrum MMP inhibitors on osteolysis associated with breast and prostate carcinoma cells [45]. These observations suggest that MMP13-selective inhibition may be an important mediator of preventing breast cancer induced bone osteolysis given its regulatory role in bone remodelling. Although the Cmpd-1 (and Zometa) delayed the development of osteolytic lesions in our models, it did not improve the overall survival of mice in the intracardiac model, such that the potential clinical utility against metastatic breast cancer seems unlikely, however combination therapy with both MMP13 inhibitors and bisphosphonates, or other systemic therapies, remains to be tested.

Indeed, we detected MMP13 expression in the tumor and osteoblasts cells in the bone metastases samples, whereas osteoclasts were largely negative for MMP13. This is in agreement with the literature reporting osteoblastic or periosteoclastic origins of MMP13 expression in mice, and its' involvement in bone collagenolysis during tumor-induced osteolysis [46], [47]. It is also consistent with the dramatic induction of MMP13 expression in cultured osteoblasts treated with the parathyroid hormone (PTH), a bone resorption-inducing agent [48], [49]. Immunolocalisation studies revealed mononucleated cells in the resorption zones that are present in close proximity of osteoclasts as a source of MMP13, and suggest its involvement in the bone resorption and matrix degradation [50], [51]. In contrast, none of the MMPs expressed by osteoclasts were found to limit the bone resorption via the direct degradation of the bone matrix [52], [53]. These findings strongly suggest that MMP13 can be selectively expressed by primary tumor and during cancer-induced osteolysis in bone metastasis.

As a comparator for any anti-osteolytic effects of Cmpd-1 we used the bisphosphonate Zometa, which is known to block osteolytic damage by inhibiting osteoclast-mediated bone resorption and also has demonstrated anti-tumor activity in preclinical models (reviewed in [54]). In contrast to Cmpd-1, mice treated with Zometa following intra-cardiac inoculation of MDA-MB-231 cells had complete abrogation of tumor-induced bone resorption, but showed no increase in overall survival. Our results contrast with data showing that Zometa, while reducing osteolytic damage in bone, has the potential to increase tumor burden in soft tissues [55]. Again, this may be a limitation of our particular isolate of MDA-MB-231 cells, which appear biased to bone metastasis, and rarely develop macroscopically visible metastases in soft tissues. It will be important, in follow-up experiments, to determine whether combination with MMP13 inhibitors will allow lower doses of Zometa to be used, since the consequences of extended Zometa use are not yet fully known, and some evidence of complications such as osteonecrosis of the jaw have been observed [56]. Although the benefit was apparent in both primary tumor growth and cancer cell induced osteolysis, suggesting that it is due to a process common to both scenarios, there was no evidence of reduced metastasis to either the lungs or brain of Cmpd-1 treated mice. This differential response to Cmpd-1 may relate to the involvement of tumor microenvironment at the orthotopic and metastatic site. Similar to Cmpd-1, there was no evidence of reduced metastasis to either the lungs or the brain of Zometa-treated mice.

Collectively, our data suggest that MMP13-selective inhibitor Cmpd-1 significantly reduces breast cancer burden at both the primary tumor site and also in bone as a secondary site, where the severity of tumor-induced bone osteolysis was also reduced. Together, these results indicate critical involvement of MMP13 in clinical breast carcinoma. MMP13 inhibition lacks MSS-like side effects, which was a major limitation in earlier trials of less specific MMP inhibitors. Combined with the lack of any apparent adverse tumor promotion due to MMP13 deficiency in MMP13-null mice, MMP13 may represent a new therapeutic target in breast cancer.
